# Observational study of the efficacy and safety of first-line osimertinib and later treatments for uncommon epidermal growth factor receptor-activating mutation-positive advanced non-small cell lung cancer

**DOI:** 10.1093/jjco/hyae176

**Published:** 2024-12-20

**Authors:** Tsuyoshi Hirata, Kageaki Watanabe, Yukio Hosomi, Kiyotaka Yoh, Kazuhiro Usui, Kazuma Kishi, Go Naka, Shu Tamano, Kohei Uemura, Hideo Kunitoh

**Affiliations:** Department of Thoracic Oncology and Respiratory Medicine, Tokyo Metropolitan Cancer and Infectious Diseases Center, Komagome Hospital, 3-18-22, Honkomagome, Bunkyo-ku, Tokyo 113-8677, Japan; Department of Thoracic Oncology and Respiratory Medicine, Tokyo Metropolitan Cancer and Infectious Diseases Center, Komagome Hospital, 3-18-22, Honkomagome, Bunkyo-ku, Tokyo 113-8677, Japan; Department of Thoracic Oncology and Respiratory Medicine, Tokyo Metropolitan Cancer and Infectious Diseases Center, Komagome Hospital, 3-18-22, Honkomagome, Bunkyo-ku, Tokyo 113-8677, Japan; Department of Thoracic Oncology, National Cancer Center Hospital East, 6-5-1, Kashiwanoha, Kashiwa, Chiba 277-8577, Japan; Department of Respiratory Medicine, NTT Medical Center Tokyo, 5-9-22, Higashigotanda, Shinagawa-ku, Tokyo 141-0022, Japan; Department of Respiratory Medicine, Toho University Omori Medical Center, 6-11-1, Ohmorinishi, Ota-ku, Tokyo 143-8541, Japan; Department of Respiratory Medicine, National Center for Global Health and Medicine, 1-21-1, Toyama, Shinjuku-ku, Tokyo 162-8655, Japan; Division of Respiratory Medicine, Department of Internal Medicine, Nihon University School of Medicine, 1-6, Kandasurugadai, Chiyoda-ku, Tokyo 101-8309, Japan; Biostatistics and Bioinformatics Course, Graduate School of Interdisciplinary Information Studies, The University of Tokyo, 7-3-1, Hongou, Bunkyo-ku, Tokyo 113-8655, Japan; Department of Biostatistics and Bioinformatics, The Interfaculty Initiative in Information Studies, The University of Tokyo, 7-3-1, Hongou, Bunkyo-ku, Tokyo 113-8655, Japan; Department of Chemotherapy, Japan Red Cross Medical Center, 4-1-22, Hiroo, Shibuya-ku, Tokyo 150-8935, Japan

**Keywords:** Osimertinib, uncommon epidermal growth factor receptor-activating mutation, non-small cell lung cancer, observational study

## Abstract

**Introduction:**

Osimertinib, a third-generation epidermal growth factor receptor (EGFR) tyrosine kinase inhibitor, is a first-line therapy for advanced or metastatic non-small cell lung cancer (NSCLC) with EGFR mutations, including both sensitizing and T790M resistance mutations. Its real-world efficacy against uncommon EGFR mutations remains under-researched.

**Methods:**

The REIWA study, a multicentric, prospective, observational study conducted in Japan from September 2018 to August 2020, enrolled patients with advanced or recurrent EGFR mutation-positive NSCLC receiving osimertinib. Data on clinical outcomes, safety, disease progression, and subsequent treatments were collected for patients with uncommon EGFR mutations.

**Results:**

Of 583 patients receiving osimertinib, 39 (6.7%) had an uncommon EGFR mutation. The present study included 32 of these patients after excluding seven patients with an exon 20 insertion mutation. The overall objective response rate was 53.1% [95% confidence interval (CI): 36.4–69.1], and the disease control rate was 78.1% (95% CI: 61.0–89.3). The median progression-free survival was 9.4 months (95% CI: 5.0–20.0), and the median overall survival (OS) was 21.8 (95% CI: 14.4–NA) months. Notably, patients with an exon21 L861Q mutation had a significantly longer OS than those with an exon18 G719X mutation, the respective values being 37.8 and 9.7 months (hazard ratio: 0.29; 95% CI: 0.10–0.85; *P* = 0.02). The rate of grade 3 or worse adverse events was 10.3%. Seven out of 32 (21.9%) patients showed progression involving only the central nervous system.

**Conclusions:**

Osimertinib demonstrated efficacy and tolerability in the clinical setting in patients with uncommon EGFR mutation-positive NSCLC.

## Teaser

Osimertinib showed efficacy and tolerability in patients with uncommon EGFR mutation-positive NSCLC, with promising response rates and survival outcomes, supporting its use as first-line therapy.

## Introduction

Epidermal growth factor receptor (EGFR)-tyrosine kinase inhibitors (TKIs) are considered the standard, first-line therapy for advanced or metastatic non-small cell lung cancer (NSCLC) harboring sensitizing EGFR mutations [1]. Osimertinib is an oral, third-generation, irreversible EGFR-TKI that selectively inhibits both sensitizing EGFR mutations and T790M resistance mutations [2].

Osimertinib has been approved for use in patients with metastatic NSCLC harboring an exon 19 deletion or exon 21 L858R mutation and for patients with a T790M resistance mutation who have demonstrated disease progression after receiving an earlier generation EGFR-TKI. The common EGFR mutations account for 75% to 80% of all EGFR mutations in NSCLC [3]. The other EGFR mutations, termed uncommon EGFR mutations, include exon 18 G719X (G719X), exon 20 S768I (S768I), exon 21 L861Q (L861Q), and exon 20 insertion (Ex 20 ins) mutations as well as complex mutations [4]. A small number of studies have reported the objective response rate (ORR) to EGFR-TKI monotherapy in patients with an exon 20 insertion mutation to be <10% [5,6]; for this reason, the guidelines do not recommend EGFR-TKI therapy as first-line therapy [7]. On the other hand, lung cancer guidelines recommend EGFT-TKIs as first-line therapy for advanced or recurrent NSCLC with an uncommon EGFR mutation other than the exon 20 insertion mutation [7].

Despite these recommendations, the efficacy and safety of osimertinib against advanced or recurrent NSCLC with an uncommon EGFR mutation have not been adequately studied. In the KCSG-LU15–09 phase II study performed in Korea, Cho *et al*. [8] reported an ORR of 50% and a median progression-free survival (PFS) of 8.2 months (median overall survival (OS) was not attained) in 36 patients harboring an uncommon EGFR mutation treated with osimertinib. In the UNICORN phase II study performed in Japan, Okuma *et al*. [9] reported an ORR of 55% and a median PFS of 9.4 months in a cohort of 40 patients. However, the real-world data on osimertinib against uncommon EGFR mutations are scarce because some of the target mutations are extremely rare. Using the data from the REIWA study, a prospective, observational study of the efficacy of osimertinib in the clinical setting, the present study evaluated the efficacy of osimertinib in patients with an uncommon EGFR mutation.

## Materials and methods

### Study design

The present, multicentric, prospective, observational study used data collection methods detailed in a previous protocol study [10]. Patients aged 20 years or older with a diagnosis of advanced or recurrent EGFR mutation-positive NSCLC who were scheduled for EGFR-TKI therapy at any of 30, participating, Japanese centers between September 2018 and August 2020 were enrolled. The clinical efficacy and safety of the treatment, exacerbation patterns, and subsequent treatments in patients receiving osimertinib were followed every six months using a case report form. This study was conducted in accordance with the Helsinki Declaration and the Japanese ethical guidelines for medical and biological research involving human subjects [11] and was approved by the ethical review committee of the Japanese Red Cross Medical Center (26 April 2019, order number 976), as well as by the relevant committee of each, participating center. Written informed consent was obtained from each patient.

### Patients

Data from the patients in the REIWA study with an EGFR mutation other than the exon 19 deletion and exon 21 L858R mutations were analyzed. A compound mutation, consisting of a common exon19 deletion or exon21 L858R mutation and an uncommon EGFR mutation, was included. The ORR to EGFR-TKI monotherapy in patients with an exon 20 insertion mutation was <10% [5,6]. Thus, EGFR-TKI monotherapy was considered ineffective as first-line therapy. Furthermore, recent trials involving uncommon mutations have excluded the exon 20 insertion mutation, for which the Japanese guidelines do not recommend the use of EGFR-TKI monotherapy [7]. Patients with this mutation were therefore excluded from the present analysis.

### Endpoints

The efficacy (response rate, PFS, OS) and safety (Grade 3 or worse adverse event rate) of the therapy, patterns of disease progression, and treatments administered after the first-line osimertinib were examined. The progression patterns were determined using the Response Evaluation Criteria in Solid Tumors (RECIST). Progressive disease (PD) was classified into nine mutually exclusive categories [10]. The progression sites were categorized as A1 [central nervous system (CNS) only, including brain metastases and carcinomatous meningitis], A2 (oligometastasis, i.e. one to three lesions in one organ other than the brain), or A3 (progression in multiple organs). The patient’s clinical condition at the time of progression was categorized as B1 (asymptomatic without clinical deterioration, i.e. PD based on radiological findings only), B2 (symptomatic but without clinical deterioration), or B3 (clinical deterioration). Clinical deterioration included decreased performance status (PS) and major, organ-threatening conditions, such as cancerous lymphangitis, bone marrow metastasis, carcinomatous meningitis, and hepatic metastasis with a hepatic disorder. This categorization produced nine (3 × 3) progression patterns.

The rate of Grade 3 or worse adverse events was graded using the National Cancer Institute Common Terminology Criteria for Adverse Events, version 5.0. PFS was defined as the period from the start of osimertinib to the date of PD, and OS was defined as the period from the start of osimertinib to death from any cause. Patients who were lost to follow-up during the observation period were classified as censored on the date of discontinuation, whereas those who did not show progression during the observation period were classified as censored on the date of the final confirmation. The final follow-up was conducted in August 2022.

### Statistical analysis

Both PFS and OS were first estimated using a univariable Cox proportional hazard model. Descriptive statistics were used to characterize the patients and the tumors. *P* < 0.05 was considered to indicate statistical significance. All statistical analyses were performed using R version 4.1.1. (R Core Team, Vienna, Austria).

## Results

Of the 583 patients in the REIWA study who received osimertinib as their first-line therapy between September 2018 and August 2020, 39 (6.7%) had an uncommon EGFR mutation.

### Patient background

Table 1 summarizes the background of the patients with an uncommon EGFR mutation. The median age was 68 years (range: 33–85 years), 21 (53.8%) were female, 16 (41.0%) had never smoked, all had adenocarcinoma, and 12 (30.8%) had a CNS metastasis. Specifically, 13 patients were L861Q-positive, 12 were G719X-positive, seven were exon 20 insertion-positive (but were excluded from the main analysis), six were T790M-positive, three were S768I-positive, and two were had some other mutation, such as L861R, etc. Two patients harbored both a L861Q and G719X mutation while two others had a S768I and G719X mutation.

**Table 1:** Baseline clinical characteristics of the patients

Characteristics	*N* = 39
Age, years	
Median	68
Range	33–85
Sex, *n* (%)	
Male	18 (46.2)
Female	21 (53.8)
Smoking status, *n* (%)	
Never	16 (41.0)
Current	3 (7.7)
Former	20 (51.2)
ECOG PS, *n* (%)	
0	12 (30.8)
1	21 (53.8)
2	4 (10.3)
3	2 (5.1)
Histological type, *n* (%)	
Adenocarcinoma	39 (100)
Overall disease classification, *n* (%)	
Metastatic	27 (69.2)
Recurrent	11 (28.2)
Locally advanced	1 (2.6)
CNS metastases, *n* (%)	
Yes	12 (30.8)
No	27 (69.2)
EGFR MT, *n* (%)^a^	
L861Q	13 (33.3)
G719X	12 (30.8)
Ex 20 ins	7 (17.9)
T790M (total)	6 (5.5)
(T790M+ exon 19 deletion)	1 (48.9)
(T790M+ L858R)	5 (45.6)
S768I	3 (7.7)
Others^b^	2 (5.1)

Abbreviations: ECOG, Eastern Cooperative Oncology Group; CNS, central nervous system; EGFR, epidermal growth factor receptor.

^a^Two patients harbored both L861Q and G719X mutations while two others had S768I and G719X mutations.

^b^“Others” includes exon21 L868R and unknown mutations.

### Clinical response and survival

**Figure 1:**
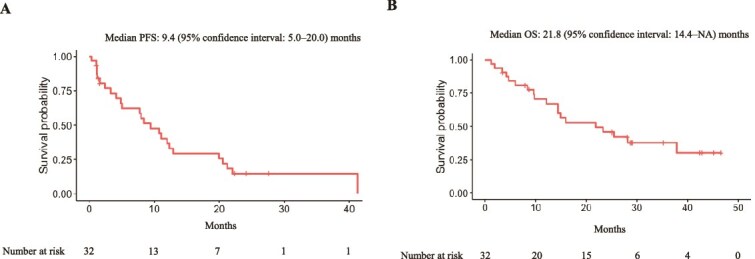
PFS and overall survival in all 32 patients. Kaplan–Meier estimates of (A) PFS and (B) OS. PFS and OS were measured from the date of osimertinib therapy initiation.

The efficacy of osimertinib was evaluated in 32 patients after excluding the seven patients with an exon 20 insertion mutation. The ORR was 53.1% (95% CI: 36.4–69.1), and the disease control rate (DCR) was 78.1% (95% CI: 61.0–89.3). The median PFS and OS for all the patients was 9.4 (95% CI: 5.0–20.0) and 21.8 (95% CI: 14.4–NA) months, respectively (Fig. 1). Of the seven patients with an exon 20 insertion mutation, five, one, and one patient had an overall response of SD, PD, and non-evaluable (NE) response. The median PFS and OS for patients with an exon 20 insertion mutation patients were 7.8 (95% CI: 3.7–NA) and 29.8 (95% CI: 6.1–NA) months, respectively.

**Figure 2:**
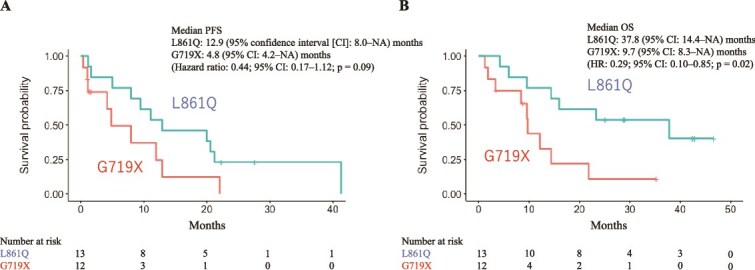
PFS and overall survival in the patients with exon21 L861Q and exon18 G719X mutations. Kaplan–Meier estimates of (A) PFS and (B) OS in the patients with exon21 L861Q and exon18 G719X mutations. PFS and OS were measured from the date of osimertinib therapy initiation.

Next, the ORR, DCR, PFS, and OS in the various mutation subgroups were evaluated to assess the efficacy of osimertinib in these subgroups (Table 2). Patients with an exon 21 L861Q mutation had a significantly longer OS than those with an exon18 G719X mutation, at 37.8 and 9.7 months, respectively [hazard ratio (HR): 0.29; 95% CI: 0.10–0.85; *P* = 0.02]. PFS was longer in patients with an exon21 L861Q mutation than in those with an exon18 G719X mutation, at 12.9 and 4.8 months, respectively although the difference was not statistically significant (HR: 0.44; 95% CI: 0.17–1.12; *P* = 0.09) (Fig. 2).

**Table 2:** Therapeutic efficacy by uncommon EGFR gene subtype

	mOS (months) 95% CI	mPFS (months) 95% CI	ORR (%) 95% CI	DCR (%) 95% CI
L861Q *n* = 13	37.8	12.9	69.2	92.3
	(14.4–NA)	(8.0–NA)	(42.0–87.6)	(64.6–100)
G719X *n* = 12	9.7	4.8	41.7	75
	(8.3–NA)	(4.2–NA)	(19.3–68.1)	(46.1–91.7)
T790M *n* = 6	28.1	10.8	66.7	83.3
	(14.9–NA)	(8.4–NA)	(29.6–90.7)	(41.8–98.9)
S768I *n* = 3	8.4	2.4	0	33.3
	(8.4–NA)	(1.1–NA)	(0–61.7)	(5.6–79.8)
All n = 32	21.8	9.4	53.10%	78.10%
	(14.4–NA)	(5.0–20.0)	(36.4–69.1)	(61.0–89.3)

Abbreviations: NA, not applicable; mOS, median overall survival; mPFS, median progression-free survival; ORR, overall response rate; DCR, disease control rate.

### Adverse events

Among the included patients, four (10.3%) had Grade 3 or worse adverse events, and four (10.3%) discontinued the treatment owing to its toxicity. Pneumonitis occurred in two patients (5.2%), although none of the cases was Grade 3 or worse.

### Progression patterns based on RECIST-PD and continuation of osimertinib after PD

PD occurred in 32 patients (82.1%) during the observation period, with A1 (CNS-only progression), A2 (oligometastasis progression), and A3 (progression in multiple organs). PD occurring in seven (21.9%), nine (28.1%), and 16 (50.0%) patients, respectively. Compared with the whole population of REIWA cohort [16], in which 37 (10.8%) developed CNS-only progression among the 344 patients in whom PD was observed, those with uncommon mutations had numerically twice as much more often CNS-only recurrences. With regard to the patient’s condition at the time of progression, B1 (asymptomatic without clinical deterioration), B2 (symptomatic but without clinical deterioration), and B3 (clinical deterioration) progressions were observed in 13 (40.6%), four (12.5%), and 15 (46.9%) patients, respectively (Table 3).

**Table 3:** Pattern of disease progression during osimertinib therapy in patients with an uncommon EGFR mutation (the numbers and percentages in the parentheses refer to the total population in the original REIWA study cohort)

Number %	Asymptomatic + no clinical exacerbation	Symptomatic + no clinical exacerbation	Clinical exacerbation^a^	Total
CNS metastasis only	4 (16)	0 (4)	3 (17)	7 (37)
	12.5% (4.7%)	0% (1.2%)	9.4% (4.9%)	21.9% (10.8%)
Oligo-progression^b^	4 (109)	2 (30)	3 (17)	9 (156)
	12.5% (31.7%)	6.3% (8.7%)	9.4% (4.9%)	28.1% (45.4%)
Multiple organs	5 (70)	2 (39)	9 (42)	16 (151)
	15.6% (20.4%)	6.3% (11.3%)	28.1% (12.2%)	50% (43.9%)
Total	13 (195)	4 (73)	15 (76)	32 (344)
	40.6% (56.7%)	12.5% (21.2%)	46.9% (22.1%)	100% (100%)

Abbreviations: CNS, central nervous system; PS, performance status.

^a^Clinical exacerbation was defined as a decline in the PS and/or exacerbation threatening major organs (carcinomatous lymphangiosis, bone marrow metastasis, carcinomatous meningitis, liver metastasis with liver damage, etc.).

^b^Single organ other than the CNS (up to three per organ).

### CNS progression

Of the total 39 uncommon EGFR mutation-positive patients treated with osimertinib, 12 (30.8%) patients had brain metastases prior to osimertinib. The EGFR mutation types in the 12 patients were L861Q (*n* = 2), G719X (*n* = 2), L861Q and G719X (*n* = 2), S768I (*n* = 1), G719X and S768I (*n* = 1), T790M (*n* = 2) and exon 20 insertion mutation (*n* = 2), respectively.

After treatment with osimertinib, eight (20.5%) patients had CNS progression, including seven (17.9%) patients had only CNS progression. Among those who had CNS progression, five (12.8%) patients experienced an exacerbation of an existing CNS metastasis, and three (7.7%) patients experienced the development of a new CNS metastasis. One patient with an exon 20 insertion mutation experienced CNS progression after osimertinib.

### Post-osimertinib therapy

Among the 30 patients with PD, 12 (40.0%) continued osimertinib. 15 patients (46.9%) received a second-line treatment after osimertinib. Platinum (carboplatin or cisplatin) plus pemetrexed (PP) was the most frequently administered therapy (*n* = 4, 26.7%). All patients who received a second-line treatment had overall response rates of 13.3% (95% CI, -2.65–31.9) and disease control rates of 46.7% (95% CI, 24.8.6–69.9). The PP group had overall response rates of 0% (95% CI, -5.6–61.7) and disease control rates of 33.3% (95% CI, 5.6–79.8).

## Discussion

The present study described the clinical result of administering first-line osimertinib to patients with an uncommon EGFR mutation. The present cohort of 32 patients had ORR of 53.1%, median PFS of 9.4 months, and median of OS 21.8 months. These findings are comparable to those of other osimertinib trials enrolling patients with uncommon EGFR mutations, such as the Korean KCSG-LU15–09 trial [8], a prospective study which reported ORR 50% and median PFS 8.2 months and the UNICORN study [9], a phase II trial conducted in Japan, which reported ORR 55.0% and median PFS 9.4 months.

Of the L861Q and G719X mutations, which were the most common mutations in the present study, the latter was associated with a poor prognosis while the former had a relatively good prognosis. Chiu *et al*. [12] reported that their cohort of 78 patients with a G719X mutation and 54 patients with a L861Q mutation had an ORR to first-generation EGFR-TKI therapy of 36.8% and 40% and a median PFS of 6.3 and 8.1 months, respectively. Yang *et al*. [13] reported that 194 TKI-naïve patients with a G719X mutation receiving afatinib therapy and 109 TKI-naïve patients with a L861Q mutation receiving the same regimen had a time to treatment failure of 14.2 and 11.5 months and an ORR of 61 and 58%, respectively. Sixteen patients with a G719X mutation (excluding those with a co-existing mutation) in the UNICORN study [14] had a 53% ORR to osimertinib and a median PFS of 8.6 months. Eleven patients in the same study with a L816Q mutation without any co-existing mutations had a 78% ORR and a median PFS of 15.7 months. In the present study, both the ORR and PFS were lower than in the previous studies. A high proportion of patients with baseline CNS metastases, along with the presence of a co-existing mutation in 33.3% of those with a G719X mutation and 16.7% of those with an L861Q mutation, may have been contributing factors. (Supplemental Table 1).

A S768I mutation was found in only three patients in the present cohort (7.7%). A large data set from a previous German study also contained only a few cases of this mutation [15], limiting our knowledge of the survival statistics.

The T790M mutation was co-expressed in six patients, all of whom also harbored a common mutation (exon 19 deletion in one patient and an L858R mutation in five patients). Our data suggested that patients with a compound mutation involving common mutations responded well to osimertinib.

The safety profile of osimertinib in this study was acceptable, in line with the findings of previous studies. However, one salient finding in our study was that pattern of disease progression analysis exhibited a higher rate of CNS metastasis than found with EGFR mutations overall, including common EGFR mutations.

The CNS-only progression rate was 21.9% for uncommon EGFR mutations and 10.8% for EGFR mutations overall [16]. To the best of our knowledge, there are no data on the progression patterns associated with uncommon EGFR mutations following osimertinib. Osimertinib was found to have high CNS penetration and activity [17]. The ORR of brain metastases in the UNICORN study [14] was 46%, a lower rate than seen in common mutations. One possible reason is that the IC50 value of osimertinib is higher in patients with an uncommon mutation [18]. The paucity of data on uncommon EGFR mutations makes it difficult to draw any conclusions, and the management of CNS metastases in patients harboring this mutation remains challenging.

The optimal approach to second-line therapy following osimertinib in patients with uncommon EGFR mutations remains unclear due to the small sample population in the present study. The limited results of this study suggest that second-line therapy following osimertinib may be less effective. As nearly all patients treated with osimertinib eventually develop resistance [19], it may be preferable to use a molecular-targeted drug tailored to the specific resistance mechanism. The findings suggested that it may be necessary to base treatment decisions on the results of a biopsy after the primary treatment to assess the resistance mechanism.

While the present study found osimertinib to be effective in patients with an uncommon EGFR mutation, the results obtained to date indicate that afatinib, a second-generation EGFR-TKI, also produces favorable results in patients with an uncommon EGFR mutation. A phase 3 trial (ACHILLES/TORG1834) comparing afatinib to chemotherapy in patients with an uncommon EGFR mutation [20] reported a median follow-up period of 12.5 months and a statistically significantly longer median PFS (10.6 months) in the afatinib group than in a chemotherapy group (5.7 months) (HR: 0.422; 95% CI: 0.256–0.694; *P* = 0.0007) [21]. The response rate was 61.4% and 47.1% and disease control rate was 82.9% and 82.4% [21] in the afatinib group and the chemotherapy group, respectively, but the difference was non-significant. Although there are no head-to-head trials comparing osimertinib with afatinib, there appears to be no remarkable difference in their efficacy. Generally speaking, afatinib is more toxic and can cause diarrhea or skin and nail toxicity, while osimertinib is more expensive [22].

This study has several limitations. As an observational study, it lacked a protocol for the use of computed tomography in evaluating treatment efficacy. Consequently, PFS may have been overestimated. Furthermore, the study focused on uncommon EGFR mutations and was statistically not powerful enough to enable different sub-groups to be compared.

## Conclusion

The efficacy of first-line osimertinib in patients with uncommon EGFR mutation-positive NSCLC in clinical practice was consistent with that reported in previous trials. Osimertinib is a viable treatment option for patients with NSCLC harboring an uncommon EGFR mutation.

## Supplementary Material

Supplementary_meterial_hyae176
