# Supplementary material for: Observational study of the efficacy and safety of first-line osimertinib and later treatments for uncommon epidermal growth factor receptor-activating mutation-positive advanced non-small cell lung cancer
Source: Jpn J Clin Oncol. 2024 Dec 20;55(3):269–74. doi: 10.1093/jjco/hyae176 (PMC11882500; doi:10.1093/jjco/hyae176)
Supplement: Supplementary_meterial_hyae176 [file supplementary_meterial_hyae176.docx]

**Supplemental Table 1.** Baseline clinical characteristics of patients with L861Q or G719X mutations

| **Characteristic** | **L861Q** | **G719X** |
| --- | --- | --- |
|  | **(N = 13)** | **(N=12)** |
| Age, years |  |  |
| Median | 66 | 68 |
| Range | 33-80 | 47-85 |
| Sex, n (%) |  |  |
| Male | 4 (30.8) | 5 (41.7) |
| Female | 9 (69.2) | 7 (58.3) |
| Smoking status, n (%) | |  |
| Never | 5 (38.5) | 3 (25.0) |
| Current | 2 (15.4) | 2 (16.7) |
| Former | 6 (46.2) | 7 (58.3) |
| ECOG performance status, n (%) | | |
| 0 | 6 (46.2) | 0 (0) |
| 1 | 5 (38.5) | 7 (58.3) |
| 2 | 2 (15.4) | 3 (25.0) |
| 3 | 0 (0) | 2 (16.7) |
| Histological type, n (%) | |  |
| Adenocarcinoma | 13 (100) | 12 (100) |
| Overall disease classification, n (%) | | |
| Metastatic | 3 (23.1) | 10 (83.3) |
| Recurrent | 10 (76.9) | 2 (16,7) |
| Locally advanced | 0 (0) | 0 (0) |
| CNS metastases, n (%) | |  |
| Yes | 4 (30.8) | 5 (41.7) |
| No | 9 (69.2) | 7 (58,3) |
| Co-mutation type, n (%) | |  |
| L861Q |  | 2 (25.0) |
| G719X | 2 (15.4) |  |
| S768I | 0 (0) | 2 (25.0) |

Abbreviations: ECOG, Eastern Cooperative Oncology Group; CNS, central nervous system; EGFR, epidermal growth factor receptor.
